# From basic mechanisms to clinical applications in heart protection, new players in cardiovascular diseases and cardiac theranostics: meeting report from the third international symposium on “New frontiers in cardiovascular research”

**DOI:** 10.1007/s00395-016-0586-x

**Published:** 2016-10-14

**Authors:** Hector A. Cabrera-Fuentes, Julian Aragones, Jürgen Bernhagen, Andreas Boening, William A. Boisvert, Hans E. Bøtker, Heerajnarain Bulluck, Stuart Cook, Fabio Di Lisa, Felix B. Engel, Bernd Engelmann, Fulvia Ferrazzi, Péter Ferdinandy, Alan Fong, Ingrid Fleming, Erich Gnaiger, Sauri Hernández-Reséndiz, Siavash Beikoghli Kalkhoran, Moo Hyun Kim, Sandrine Lecour, Elisa A. Liehn, Michael S. Marber, Manuel Mayr, Tetsuji Miura, Sang-Bing Ong, Karlheinz Peter, Daniel Sedding, Manvendra K. Singh, M. Saadeh Suleiman, Hans J. Schnittler, Rainer Schulz, Winston Shim, Daniel Tello, Carl-Wilhelm Vogel, Malcolm Walker, Qilong Oscar Yang Li, Derek M. Yellon, Derek J. Hausenloy, Klaus T. Preissner

**Affiliations:** 1 Institute of Biochemistry, Medical School, Justus-Liebig University, Giessen, Germany; 2 Cardiovascular and Metabolic Disorders Program, Duke-National University of Singapore, 8 College Road, Singapore, 169857 Singapore; 3 National Heart Research Institute Singapore, National Heart Centre Singapore, Singapore, Singapore; 4 Department of Microbiology, Kazan Federal University, Kazan, Russian Federation; 5 Centro de Biotecnología-FEMSA, Tecnológico de Monterrey, Monterrey, NL Mexico; 6 Research Unit, Hospital of Santa Cristina, Research Institute Princesa, Autonomous University of Madrid, Madrid, Spain; 7 Department of Vascular Biology, Institute for Stroke and Dementia Research, Klinikum der Ludwig-Maximilians-Universität, Munich, Germany; 8 Munich Cluster for Systems Neurology (SyNergy), Munich, Germany; 9 Department of Cardiovascular Surgery, Medical School, Justus-Liebig-University, Giessen, Germany; 10 Center for Cardiovascular Research, John A. Burns School of Medicine, University of Hawaii, Honolulu, USA; 11 Department of Cardiology, Aarhus University Hospital, Skejby, Aarhus N, Denmark; 12 Department of Biomedical Sciences, University of Padova, Padua, Italy; 13 Experimental Renal and Cardiovascular Research, Department of Nephropathology, Institute of Pathology, Friedrich-Alexander-Universität Erlangen-Nürnberg, Nuremberg, Germany; 14 Institut für Laboratoriumsmedizin, Ludwig-Maximilians-Universität, Munich, Germany; 15 Institute of Human Genetics, Friedrich-Alexander-Universität Erlangen-Nürnberg, Nuremberg, Germany; 16 Department of Pharmacology and Pharmacotherapy, Semmelweis University, Budapest, Hungary; 17 Pharmahungary Group, Szeged, Hungary; 18 Department of Cardiology, Sarawak Heart Centre, Sarawak, Malaysia; 19 Institute for Vascular Signalling, Centre for Molecular Medicine, Goethe-University, Frankfurt, Germany; 20 D. Swarovski Research Lab, Department of Visceral, Transplant Thoracic Surgery, Medical Univ Innsbruck, Innsbruck, Austria; 21 Department of Cardiology, Dong-A University Hospital, Busan, Korea; 22 Hatter Institute and MRC Inter-University Cape Heart Unit, Faculty of Health Sciences, University of Cape Town, Cape Town, South Africa; 23 Institute for Molecular Cardiovascular Research, RWTH University Hospital, Aachen, Germany; 24 Department of Cardiology, The Rayne Institute, St Thomas’ Campus, King’s College London, London, UK; 25 The James Black Centre, King’s College, University of London, London, UK; 26 Department of Cardiovascular, Renal and Metabolic Medicine, Sapporo Medical University School of Medicine, Sapporo, Japan; 27 Baker IDI Heart and Diabetes Institute, Melbourne, Australia; 28 Department of Cardiology and Angiology, Hannover Medical School, Hannover, Germany; 29 Bristol Heart Institute, University of Bristol, Bristol Royal Infirmary, Bristol, UK; 30 Institute of Anatomy and Vascular Biology, Westfalian-Wilhelms-University, Münster, Germany; 31 Institute of Physiology, Justus-Liebig University, Giessen, Germany; 32 Department of Pathology, John A. Burns School of Medicine, University of Hawaii, Honolulu, USA; 33 The Hatter Cardiovascular Institute, University College London, London, UK; 34 The National Institute of Health Research University College London Hospitals Biomedical Research Centre, London, UK; 35 Department of Cardiovascular Medicine, National Institute of Cardiology, Ignacio Chavez, Mexico, D.F., Mexico

**Keywords:** Cardiomyocyte signaling pathways, Cardioprotection, Cardiovascular disease, Co-morbidities, Drug targeting, Endothelial permeability, Extracellular RNA (eRNA), Heart regeneration, Induced pluripotent stem cells, Ischemia–reperfusion injury, Lipid metabolism, MicroRNAs (miRNAs), Mitochondria, Remote ischemic conditioning

## Abstract

In this meeting report, particularly addressing the topic of protection of the cardiovascular system from ischemia/reperfusion injury, highlights are presented that relate to conditioning strategies of the heart with respect to molecular mechanisms and outcome in patients’ cohorts, the influence of co-morbidities and medications, as well as the contribution of innate immune reactions in cardioprotection. Moreover, developmental or systems biology approaches bear great potential in systematically uncovering unexpected components involved in ischemia–reperfusion injury or heart regeneration. Based on the characterization of particular platelet integrins, mitochondrial redox-linked proteins, or lipid-diol compounds in cardiovascular diseases, their targeting by newly developed theranostics and technologies opens new avenues for diagnosis and therapy of myocardial infarction to improve the patients’ outcome.

## Introduction

Despite the progress in our understanding of cellular signaling mechanisms in cardiomyocytes and the cellular communication within the cardiovascular system as well as new treatment options for cardiovascular diseases, ischemic heart disease remains the leading cause of death and disability worldwide [14, 30, 32, 40, 44, 71, 72]. Usually, such events are manifested by acute thrombotic occlusion of a main coronary artery at preselected sites of disturbed blood flow or at ruptured atherosclerotic lesions [18, 56]. Although percutaneous coronary intervention (PCI) is the treatment of choice for reducing the size of a myocardial infarction (MI), the induced reperfusion may not only lead to the recovery of ischemic cardiac tissue but also brings about the paradoxical phenomenon of myocardial “ischemia/reperfusion injury” (IRI).

During the recent 3rd International Symposium on “New Frontiers in Cardiovascular Research” (Singapore), basic researchers and clinicians discussed new biomedical developments as well as new players in cardiovascular diseases, novel targets, and respective interventional strategies, particularly in the area of heart failure. The same holds true for mechanisms of action of the emerging class of atypical chemokines that was focused on at the symposium. Based on novel methods using single-chain antibodies, induced pluripotent stem cells, or miRNAs, novel drugs and technologies, summarized as “Theranostics”, were presented and heavily discussed. In essence, the meeting covered heterogenous and unrelated intra- as well as extracellular molecular targets, which are all linked to the development or prevention of cardiovascular diseases, not only reflecting the complexity of the biological system but also indicating the variety of possible interventional approaches that can be helpful or even lifesaving as cardioprotective strategies.

## Ischemia/reperfusion injury and cardiac conditioning: basic mechanisms

While myocardial IRI is associated with an increased death of cardiomyocytes, brief cycles of ischemia and reperfusion (termed “ischemic conditioning”) appear to protect the heart from acute MI and IRI [5, 27, 72]. This phenomenon was discovered 30 years ago and stimulated intense research and clinical trials to understand the mechanisms of myocardial IRI and cardioprotection in patients presenting with ST elevation MI or undergoing cardiac surgery. Yet, experimental animal models as well as clinical studies have presented diverging results of ischemic conditioning in cardiac surgery and PCI or interventions following acute MI [27, 28, 38, 41, 72]. IRI can manifest as reperfusion-induced arrhythmias, myocardial stunning, microvascular obstruction, and cardiomyocyte death, being the main cause of reperfusion-induced myocardial lethality. While the former two situations are more or less self-terminating, the third situation occurs in more than 50 % of patients and is associated with capillary damage, microthrombosis, and cardiomyocyte swelling. Moreover, oxidative stress, calcium overload, and irreversible hypercontracture of cardiomyocytes contribute to lethal myocardial IRI [33, 35]. Myocardial IRI has been investigated in experimental animals, and attenuation of this injury can be obtained by pharmacological and mechanical conditioning strategies, including ischemic pre- and postconditioning and “remote ischemic conditioning” (RIC) [36, 45].

The characterization of the underlying mechanisms and the contributing components in RIC will be a major challenge for future translational work in the cardiovascular field to reduce infarct size and improve clinical outcomes in patients with ischemic heart disease [41, 80]. During tissue injury and upon exposure of tissue towards hypoxia as a source of tissue stress, large amounts of intracellular components as alarming compounds are released that disseminate into the circulation or remain at the site of cell activation/damage and trigger inflammation by activating the innate immune system [109]. In particular, nuclear proteins, such as histones, heat-shock proteins, or amphoterin [56, 79], as well as nuclear DNA [99], mitochondrial DNA [7, 103, 110], ribosomal RNA [86, 87, 111], and miRNAs, become liberated in isolated or complexed form, such as in association with exosomes or microvesicles [14, 15, 17, 25]. The innate immune system senses the signals released by the necrotic cells and activates inflammatory pathways to neutralize such danger signals [75].

In this regard, the endogenous extracellular RNA/RNase system appears to provide new alarmins, primarily present following tissue stress (such as in hypoxia) and cell damage: extracellular RNA (eRNA), together with Tumor Necrosis Factor α (TNF-α), serve as damaging factors in experimental IRI, whereas adminstration of RNase1 had a major therapeutical impact by significantly reducing the infarct area and preventing cardiomyocyte death [16, 17]. Moreover, in a small pilot clinical study, patients undergoing cardiac surgery received initial RIC (4 × 5 min limb ischemia) or a sham procedure. The RIC protocol significantly increased plasma levels of endogenous vascular RNase1 (derived from endothelial cells) with the consequence that circulating eRNA (from arterial and coronary sinus blood) became substantially decreased [15]. Moreover, in rats undergoing the RIC procedure, RNase1 significantly rose in animals receiving buprenorphine or isoflurane, when compared with RIC without these drugs, implying a significant contribution of the RIC-dependent endothelial RNase1 release for improving the outcome of cardiac IRI. Yet, the exact mechanism of RNase1-induced cardioprotection still remains to be uncovered, although split products of RNA, such as nucleotides or nucleosides, may be valuable candidates to confer cardioprotection. It is also possible that medical preconditioning exhausts the conditioning capacity of mammalians, such that RIC may not promote additional preconditioning anymore.

“Macrophage migration inhibition factor” (MIF) family proteins feature overlapping properties with protein-type alarmins, such as high mobility group binding protein-1 (HMGB-1) or RNase1. MIF is known to be a pleiotropic inflammatory cytokine with chemokine-like functions and as such is a prototypical member of the emerging class of atypical or chemokine-like function proteins [95]. MIF is also a major pro-atherogenic factor, but counter-intuitively exhibits protective activities in the heart, damaged by IRI [6, 65]. Interestingly, the cardioprotective effects of MIF are predominant in the early reperfusion phase [74] and are amplified by post-translational modifications, such as S-nitrosylation and N-oxidation. In line with these observations, exogenously administered (“unmodified”) recombinant MIF was unable to convey RIC in a Langendorff heart model [78]. Moreover, MIF produced in the later phase and exacerbated IRI through promoting inflammatory leukocyte infiltration and activation [20, 76]. The role of novel MIF family member MIF-2/D-DT is currently unclear, as its knockout in a mouse model led to an exacerbation of infarct size, while its levels in patients undergoing coronary artery bypass grafting are predictive of ischemia–reperfusion outcome parameters, such as acute kidney injury [92], clearly necessitating the application of additional models on this and to-be-discovered family members, as well as on the MIF/MIF-2 double knockout mouse.

## Ischemia/reperfusion injury and cardiac conditioning: clinical applications

The translation of the RIC procedure to patients has been challenging, because improvement of clinical outcome has been observed only in a minority of studies with RIC and findings are not consistent [30, 41, 80]. The explanation for the difficulties in translating the beneficial findings in experimental animals to humans remains unclear and may include confounding factors, such as age, co-morbidity, co-medication and anesthetic regimen in procedures requiring general anesthesia [10, 23, 49]. The intensity of the conditioning procedure also seems important and depends on the number and duration of the conditioning cycles, thereby defining the efficacy of protection by a specific algorithm [36, 45]. The indicated intervention can be initiated already in the ambulance during transportation to the PCI table and increases myocardial salvage leading to improved left ventricular function and outcome when used as an adjunct to primary PCI [90]. In the clinical setting, RIC was found to be effective in patients with diabetes mellitus and other cardiovascular risk factors as well [91, 105].

Yet, in two recent multicentre double-blind randomised controlled clinical trials (RIP-Heart, ERICCA) [64, 105], neutral results were reported upon application of RIC in patients, whereby the type of pre-operative medication appears to have a major impact on the outcome as well [29, 37]. Interestingly enough, both neuronal and vascular factors seem to play an important role in promoting RIC, as demonstrated in experimental animal and human studies [36, 57, 73]. Nevertheless, interventional cardiological procedures in acute angioplasty for ST-segment elevation MI seem to hold the best potential at present.

Despite the successful basic and clinical ischemic-reperfusion research for three decades, no definitive cardioprotective drugs are available yet. The lack of successful translation of experimental results into the clinical setting may be due to (1) the lack of proper co-morbidity experimental models as well as (2) the existence of hypothesis-driven-biased approaches to find molecular targets. Indeed, major cardiovascular co-morbidities, such as hypertension, hyper-lipidemia, diabetes, and their co-medications, interfere with most of the known cardioprotective mechanisms [23]. Moreover, cardiovascular co-morbidities as well as the available conditioning procedures affect the global myocardial gene expression profile at the transcriptional level and the fine-tuning regulators of translation, such as miRNAs [97, 98]. Thus, the comprehensive analysis of the cardioprotective gene expression fingerprint at the transcription and protein level in normal, protected, and in co-morbid probands may lead to the identification of novel molecular targets for cardioprotection by an unbiased non-hypothesis-driven approach.

**Fig. 1:**
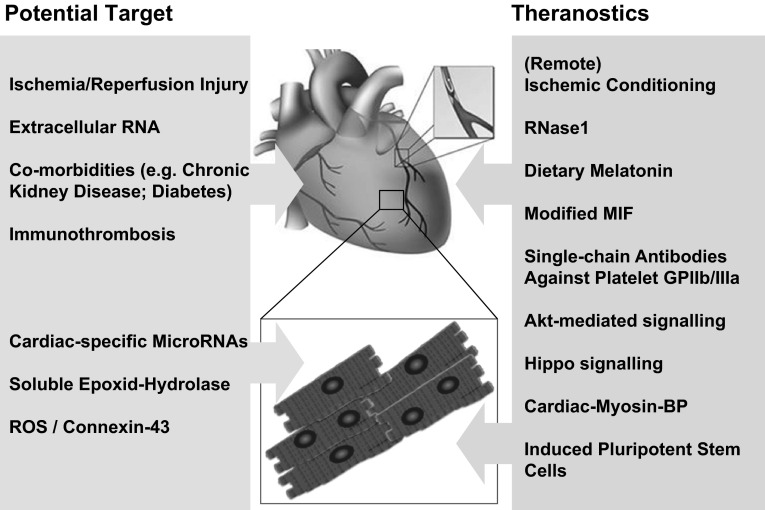
Potential new targets and theranostics in cardio-protection. The basic mechanisms, preclinical models and some clinical applications of several cardio-destructive pathologies and cardiovascular diseases (*red box*) are discussed in the text. Existing and novel antagonistic procedures as well as the related theranostics (*green box*), both in vitro and in experimental models, were found to promote cardio-protection on different molecular levels, particularly improving the functional status of cardiomyocytes (*ROS* reactive oxygen species, *MIF* macrophage migration inhibition factor, *GP* glycoprotein, *BP* binding protein)

Like other co-morbidities, chronic kidney disease (CKD) is known as a major risk factor of cardiovascular events and mortality after MI [104]; yet, the contribution of CKD to myocardial IRI remains unclear. In systematic studies, the influence of CKD on cytoprotective signaling was analyzed using a rat model of CKD, particularly addressing the functional role of Akt-phosphorylation. The results indicate that CKD suppresses Akt-activation upon reperfusion with the disruption of protective signaling to mitochondria, associated with infarct size enlargement. Moreover, the impact of CKD on infarct size depends on the severity of CKD that is directly related to insufficient activation of Akt at the time of reperfusion. Chronic treatment with an erythropoietin receptor ligand prevented CKD-induced enlargement of myocardial infarct size by restoration of Akt-mediated signaling possibly via normalized malate-aspartate shuttle flux in cardiomyocytes [69]. These promising data may provide clues for deciphering other co-morbidities and their mechanistic relations to the metabolic status in the context of cardiovascular diseases (Fig. 1).

With regard to the multi-ethnic population in a developing country like Malaysia, a pool of cardiac patients was drawn from both urban and rural areas for the analysis of acute coronary syndrome, being the leading cause of mortality. A striking feature was found in that the majority of patients were presented at a younger age group compared with similar populations in developed countries. While early intervention for ST-elevation MI has more relevance in urban areas with well-staffed care centres [96], substantial data from the voluntary “National Cardiovascular Disease Registry” (launched in 2006 in Malaysia) resulted in the improvement of risk stratification mechanisms for patients from rural areas [3]. Such strategies appear to serve as a role-model and can be relevant to the population at risk in other developing countries as well.

## New players in cardiovascular diseases

The integrity and functionality of the monolayer vascular endothelium as a lively and dynamic barrier between the flowing blood and the underlying tissues, including the heart, depend on the natural implementation of distinct, ultralarge protein complexes at cell–cell borders, characteristic for adherens-, tight- und gap-junctions [81]. Newly described, highly dynamic structures, termed “Junction-associated intermittent lamellipodia” (JAIL), which are controlled by the WAVE-WASP/ARP2/3 protein complex, are driven by actin filament rearrangement to provide small plasma membrane protrusions that preferentially appear at junctional sites of endothelial cells with a low level of cadherin-5 (VE-cadherin) [1, 2, 84]. Thrombin as a pro-inflammatory mediator blocks JAIL formation and thus increases endothelial permeability. Such dynamic processes were made visible by stimulated emission depletion (STED) fluorescence microscopy and 3D reconstruction “structured illumination microscopy” (SIM). Since these structures are also disturbed by fluid shear stress and other stimulants, such as hypoxia, it remains to be analyzed in which way JAIL plays a role in, e.g., ischemia-driven disintegration of the endothelium in small and large cardiac vessels or during myocardial IRI.

An emerging new concept relates to innate immunity-related protective mechanism of the heart. In the context of ischemic heart disease, TNF-α, a major player of the immune system, initiates the induction of a cardioprotective signaling pathway [89] that involves the activation of the signal transducer and activator of transcription 3 (STAT-3) [70], designated as the SAFE (“Survivor Activating Factor Enhancement”) pathway [50, 53]. Toll-like receptor 4 (TLR4), sphingosine-1 phosphate, and activation of specific miRNAs are involved in this pathway as well [48]. In particular, TLR4 may trigger the activation of the SAFE pathway to promote cell survival following myocardial IRI [68]. Dietary melatonin, given at a concentration found in red wine, was demonstrated as respective trigger to confer cardioprotection [52] but also to prevent pulmonary hypertension via the activation of the SAFE pathway [59]. Based on the fact that high-density lipoproteins (HDL) can also activate the SAFE pathway, sphingosine-1 phosphate was identified as predominant component of HDL to protect against myocardial IRI [94], ultimately regulating the mitochondrial functions of cardiomyocytes [13]. With the detailed analysis of various lipoprotein subfractions (Lipoprint^®^), new insights into their composition and functionality in patients suffering from cardiovascular diseases are now available, and this may provide a personal cardiac risk calculator.

So far, microvascular obstruction has been linked to many vascular diseases, including stroke, myocardial infarction, thrombotic microangiopathies, infections, and cancer [39, 85]. Although the pathogenetic relevance of microangiopathies, such as haemolytic-uremic syndrome or disseminated intravascular coagulation, during sepsis has clearly been demonstrated, the pathogenetic mechanism of microvascular thrombosis has remained enigmatic. It has been assumed that the failure to assign a clear pathogenetic role to microvascular thrombosis in many diseases is due to difficulties in its detection and in the inability to assess the efficacy of antithrombotic treatments in the clinical situation. It has recently been shown that during systemic bacterial infections, microvascular thrombosis under certain conditions acts as an instrument of intravascular immunity [22, 62]. In organs, such as the liver and spleen, fibrin-rich microthrombi support the containment and elimination of *Escherichia coli* inside blood vessels and thereby prevent tissue invasion and dissemination of the pathogens. This mechanism has been termed “Immunothrombosis”. Immunothrombosis is suggested to form a major biological basis of pathological microvascular and macrovascular thrombosis (especially deep vein thrombosis), together with the physiological mechanism arresting bleeding (haemostasis). Immunothrombosis is a transient process as it appears to be normally resolved within 2 days. Pathological forms of microvascular thrombosis during infections, such as disseminated intravascular coagulation, are likely caused by an excessive activation of immunothrombosis and/or by its impaired resolution [82]. Most probably, the formation of microvascular thrombi under non-infectious conditions might equally be able to protect the intravascular compartment from damage as caused by immune complexes, circulating cell fragments, or endothelial damage. This would indicate that the beneficial nature of microvascular thrombosis may also apply to non-infectious conditions. Hence, the failure to document a pathological role for microvascular thrombosis under several pathological conditions could potentially, at least in part, be related to the fact that it is host-protective under those conditions.

Following atherectomy, drug-eluting stents allow a defined local application of anti-proliferative agents and other drugs to reduce neointimal formation and restenosis. Despite the tremendous success of drug-eluting stents using unselective cytostatic substances, their efficacy and safety need further improvement. However, strategies to achieve both of these goals are currently lacking. Likewise, myocardial remodeling and regeneration rely on myocardial capillary density and thus on effective neovascularization after MI. Yet, the mechanisms underlying myocardial angiogenesis and its regulation by miRNAs are not well defined. Comprehensive screens were established to analyze the expression of non-coding RNAs during the development of neointimal hyperplasia in established mouse models of atherogenesis, and particular miRNAs that are regulated in a lesion-, time- and cell-specific manner were identified. As an example, inhibition of miRNA-92a appeared to be safe as an effective treatment to prevent neointima formation as well as to improve re-endothelialization at the same time [19]. In addition, miRNA-146a was upregulated in the ischemic myocardium of mice following ligation of the left anterior descending artery in a time-dependent manner. In vitro, the overexpression of miRNA-146a significantly attenuated endothelial cell proliferation, migration, and abolished endothelial capillary network formation. In contrast, knock-down of miRNA-146a markedly augmented endothelial cell proliferation, migration, network formation, and sprouting (unpublished data). Mechanistically, NOX4, NOTCH1, and nRAS were identified and validated as direct targets of microRNA-146a in endothelial cells (unpublished data). In vivo, antagomirs against miRNA-146a significantly enhanced angiogenesis and re-vascularization in the infarcted myocardium, accompanied by preserved cardiac function and a markedly reduced infarct size (unpublished data). It is expected that additional cardiac-specific miRNAs become characterized that would serve as attractive targets for future therapeutic interventions in the treatment of ischemic heart disease.

Besides the well-known bioactive lipid mediators derived from arachidonic acid by cytochrome-P450-catalyzed reactions, other polyunsaturated fatty acids can be metabolized to epoxides and then diols by the actions of cytochrome P450 enzymes followed by soluble epoxide hydrolase (sEH). These metabolites appear to be ignored in their physiological relevance in the cardiovascular system, although they become generated in high concentrations. Deletion of sEH significantly delayed angiogenesis in the retina, a phenomenon associated with activation of the Notch signaling pathway [43]. sEH-deficient mice are also largely protected against the development of type 2 diabetes and the associated hypertension when fed a high fat diet. This occurs at the expense of the liver, as sEH controls the expression of key enzymes involved in lipid metabolism. Thus, inhibitors of sEH that increase epoxide, but decrease diol levels have potential for the treatment of the metabolic syndrome/type 2 diabetes (influencing cholesterol homeostasis) and its cardiovascular complications [60]. In which way the level of sEH may affect the processes during myocardial IRI as they relate to the disturbance of vascular integrity or the dysfunction of cardiomyocytes remains to be studied (Fig. 1).

## Cardiac repair and regeneration

The understanding of developmental and regenerative processes of heart biology and cardiomyocyte proliferation and the underlying mechanisms may lead to their reactivation in diseased hearts postnatally and may have a great potential to protect or improve heart function. It is known that the mammalian heart loses its ability to regenerate, largely due to the fact that after birth cardiomyocytes fail to undergo cytokinesis. Instead, they exit the cell cycle after karyokinesis resulting in bi-nucleated cells, and cardiac tissue further expands by hypertrophy. Moreover, the efficiency in inducing cardiomyocyte proliferation decreases proportionally to cardiomyocyte age [54, 107]. Thus, an understanding how cardiomyocyte cytokinesis is regulated during development might provide new clues towards cardiac regeneration. Interestingly enough, cardiomyocyte centrosome integrity is lost shortly after birth in mammals. This is coupled with the relocalization of various centrosome proteins to the nuclear envelope. Consequently, postnatal cardiomyocytes are unable to undergo ciliogenesis, and the nuclear envelope adopts the function as cellular microtubule organizing center [108]. Loss of centrosome integrity is associated with, and can promote, cardiomyocyte G0/G1 cell cycle arrest, suggesting that centrosome disassembly is developmentally utilized to achieve the post-mitotic state in mammalian cardiomyocytes. In contrast, adult newt and zebrafish maintain the ability to regenerate their hearts through proliferation of cardiomyocytes, which also retain centrosome integrity. Based on these novel results, underlying the post-mitotic state of mammalian cardiomyocytes, potential mechanisms of heart regeneration in zebrafish and newts are testable that may provide clues for the regeneration of mammalian hearts. In addition, using systems biology approaches [24], novel regulators of cardiac development and regulatory networks were identified, integrating large-scale expression datasets. As an example, the joint analysis of a high-resolution temporal expression data set describing heart development and a transcriptomic dataset describing induced cardiomyocyte proliferation were merged and are currently being experimentally validated, eventually leading to novel candidate cytokine genes for cardiac regeneration.

Hippo signaling has been implicated in cardiac development and regeneration after myocardial injury. Genetic deletion of upstream Hippo signaling kinases (*Mst1/2*, *Lats2,* or *Salvador*) leads to an expansion of ventricular myocardium due to increased cardiomyocyte proliferation [31]. Global deletion of *Yap* results in the early embryonic lethality due to defects in multiple tissues, including yolk sac vasculogenesis, chorioallantoic fusion, and body axis elongation. However, *Taz* mutant mice are viable but develop glomerulocystic kidney disease and pulmonary disease. Genetic deletion of Yap and Taz both leads to the early embryonic lethality suggesting functional redundancy. Despite the studies described above, a role for Yap and Taz in the epicardium during coronary vasculature development has not been explored. Formation of the coronary vasculature is a complex and precisely coordinated morphogenetic process that begins with the formation of epicardium. The epicardium gives rise to many components of the coronary vasculature, including fibroblasts, smooth muscle cells, and the endothelium. While Hippo signaling mediators Yap and Taz are expressed in proepicardial and epicardial cells, a combination of genetic and pharmacological approaches that inhibited Hippo signaling mediators Yap and Taz also impaired epicardial epithelial-to-mesenchymal transition (EMT) as well as a reduction in epicardial cell proliferation and differentiation into coronary endothelial cells. As a conclusion, Yap and Taz control epicardial cell behavior, in part by regulating *Tbx18* and *Wt1* expression. These findings show a role for Hippo signaling in epicardial cell proliferation, EMT, and cell fate specification during cardiac organogenesis [88].

## Cardiac mitochondria and cardioprotection

Reactive oxygen species (ROS) at high levels do play an adverse role in myocardial IRI, but contribute to endogenous cardioprotection at lower concentrations [35]. Moreover, the aforementioned conditioning protocols appear to recruit complex signaling cascades of activation of cardiomyocyte sarcolemmal receptors, intracellular enzymes, as well as ROS and nitrosative species, to gain mitochondrial stabilisation and finally to protect against cell death. The following questions remain to be addressed: What are the relevant sources of ROS in the cytosol and the mitochondria of cardiomyocytes? Which proteins/enzymes do contribute to ROS formation at different levels and how does the ROS-induced ROS release work in detail? A strong case can be made for the importance of the gap junction protein connexin 43 in the context of myocardial IRI and cardioprotection. Apart from being present at gap-junctions along cardiomyocyte cell borders, connexin 43 is also located at mitochondria and is involved in mitochondrial respiration, ATP generation, and mitochondrial potassium influx [8, 9, 11, 12, 55, 67, 83]. Blockade of connexin 43-formed channels reduces myocardial IRI, but, at the same time, also abolishes cardioprotection induced by ischemic preconditioning. Another redox-linked protein is p66SHC, which translocates into mitochondria where it catalyzes electron transfer from cytochrome c to oxygen resulting in ROS production [34]. Deletion of p66SHC, however, does not reduce infarct size in mice in vivo undergoing 30 min ischemia and 120 min reperfusion (unpublished data), but p66SHC contributes to vascular abnormalities related to diabetes and aging. On the other hand, ROS formation might contribute to self-endogenous defense against mild myocardial IRI [21], whereas p66SHC knockout does not affect endogenous cardioprotection (unpublished data).

It has been shown that the mitochondrial protein NDUFA4L2 plays an essential role in decreasing oxygen consumption and ROS production through inhibition of respiratory chain-complex I [93]. However, even though NDUFA4L2-induced mitochondrial repression has been proven by several research groups [51, 66], its physiological role remains unknown. It appears that the “hypoxia-inducible factor” (HIF)-NDUFA4L2 axis acts as one of the major pathways for cellular adaptation towards hypoxia via mitochondrial activity suppression. Along this line, NDUFA4L2 protein was highly expressed in heart tissue, whereby the cardiac fetal tissues exhibited highest levels when compared with tissues of adult mice. Finally, since the fetal heart is one of the sites that present higher levels of NDUFA4L2, a specific cardiac knockout mouse line was successfully generated by breeding a conditional NDUFA4L2 exon 2-floxed mice line with a with a heart-specific CRE (Nkx 2.5) transgenic line. However, these mice are not embryonically lethal and the role of cardiac NDUFA4L2 in adulthood warrants further investigation.

## Diagnosis of cardiac damage and new theranostics

The diagnosis of “Non-ST elevation myocardial infarction” (NSTEMI) is dominated by the need to document an elevation in cardiac troponins I or T above the population-defined 99th centile. Yet, these biomarkers are released only slowly, thereby delaying the rule-in or the rule-out criteria for NSTEMI. The latest ESC guidelines attempt to circumvent this obstacle by adopting a ‘rule-out’ troponin value significantly below the 99th centile and a ‘rule-in’ value well above the 99th centile [77]. However, this leaves the majority of patients in an undefined diagnostic window, requiring repeated testing and further observation. A new cardiac biomarker, the “Cardiac myosin-binding protein C” (cMyC), had been recently introduced, which constitutes an abundant sarcomeric protein with a unique cardiac isoform that is released upon cardiac damage or iatrogenic MI much faster than the troponins [4, 26]. Using newly generated highly specific monoclonal antibodies, an ultrasensitive assay to measure cMyC in biological samples was established with a detection limit of 0.4 ng/l and intra- and inter-series precision around 10 % [61]. In ambulatory patients, cMyC concentration in plasma (median 12.23 ng/l) was linearly correlated with troponin levels, and both parameters showed a similar dependence on age, renal function, or left ventricular activity. In another patient cohort with aortic stenosis, cMyC levels were strongly related to fibrosis (detected by cardiac magnetic resonance imaging) and clinical outcome. It is expected that cMyC will be introduced into the clinics as new diagnostic parameter, e.g., after spontaneous, type 1 MI [46] as well as in other groups of cardiac patients to obtain fast and reliable results on the degree of cardiac damage.

Besides diagnostic markers, there is also need for new prognostic biomarkers. With the rise in obesity and its associated metabolic complications, many more patients will be considered at high risk of cardiovascular disease. Circulating miRNAs have recently evolved as novel players in the field of medicine [102]. Platelets contain and release miRNAs and are a major source of abundant miRNAs in plasma and in serum. There is a striking correlation of miRNAs with platelet activation markers in the general population and platelet-derived miRNAs in plasma correlate with indices of platelet function in patients on dual anti-platelet therapy [47]. Moreover, platelet miRNAs appear to alter their function, most probably by influencing gene expression in megakaryocytes [47]. Since thrombus formation is a key event in triggering the clinical manifestations of atherosclerotic disease, it could be informative to assess platelet activation in the context of cardiovascular risk. Similarly, circulating angiogenic miRNAs have been linked to the onset and progression of retinopathy in patients with type 1 diabetes [106].

To provide an efficient antithrombotic therapy in the context of cardiovascular diseases, the detection and elimination of thrombi and emboli are the major challenge in clinical practice. Here, the site-directed molecular imaging and the associated therapeutic targeting of platelet-specific antigens is a highly promising approach. An established target in the therapy of cardiovascular disease is the integrin αIIb-β3 (GP IIb/IIIa, CD41/CD61), the highly abundant fibrinogen receptor on the activated platelet surface. Since this integrin undergoes a conformational change upon platelet activation, the exposed characteristic epitopes can be used as thrombus-specific targets. Following the screening by phage-display of PCR-cloned human single-chain antibodies, highly specific integrin antagonists were generated. These were coupled by genetic, chemical, or biological approaches to obtain various fusion products, which are available either as therapeutic drugs or in combination with contrast particles. Using these approaches, various anticoagulants, anti-platelet drugs, or fibrinolytics were specifically targeted to the thrombus site to achieve effective thrombolysis and prevention of emboli without any bleeding complications in mice [42]. Moreover, these procedures allow the design of targeted “theranostic compounds”, such as ultrasound micro-bubbles, magnetic resonance nanoparticles, or positron emission tomography tracers, for thrombus detection with high sensitivity and specificity in the respective imaging modality [101]. Compounds are underway that allows the combination of detection and imaging of thrombi with concomitant effective treatment together with the monitoring of success or failure of therapy [100] (Fig. 1).

Human-induced pluripotent stem cell (iPSC)-derived cardiomyocytes present a tremendous opportunity for the study of cardiac arrhythmias in vitro. The characterization of cellular models for major subtypes of inherited channelopathy revealed that these defects were caused by dysfunctional potassium and sodium channels that contribute to the Long QT Syndrome (LQTS) 1, LQTS2, and LQTS3 [63]. In particular, the correction of the trafficking of KCNH2 (LQTS2) potassium channel through intracellular mechanisms restored hERG currents and reduced arrhythmia in LQTS2 patient-derived cardiomyocytes, also documenting the usefulness of iPSC-cardiomyocytes in LQTS2 modeling and drug testing. Moreover, iPSC-cardiomyocytes were used as a tool to evaluate the cardiac toxicity of topical drugs [58]. These applications document the powerful iPSC technology as value creation for understanding the pathomechanisms of cardiac arrhythmias, but also for drug testing and toxicology research.
